# Transmission Line Sag Measurement and Simulation Research Based on Non-Contact Electric Field Sensing

**DOI:** 10.3390/s22218379

**Published:** 2022-11-01

**Authors:** Jinhua Zuo, Jing Fan, Yong Ouyang, Hua Liu, Chao Yang, Changjin Hao

**Affiliations:** 1 School of Electrical and Information Technology, Yunnan Minzu University, Kunming 650500, China; 2 University Key Laboratory of Information and Communication on Security Backup and Recovery in Yunnan Province, Kunming 650500, China; 3 Tsinghua Sichuan Energy Internet Research Institute, Chengdu 610213, China

**Keywords:** sag measurement, transmission line, electric field sensing, electric field measurement, sensor arrays, non-contact monitoring

## Abstract

Sag is an important indicator of the operational health of a transmission line, and its timely measurement is of great significance to maintain the stability and reliability of power systems. However, traditional contact measurements may be affected by the electromagnetic interference of conductors. In contrast, measurement methods without direct electrical contact with the subject provide greater portability and flexibility. This paper presents a study of a transmission line sag measurement and simulation based on non-contact electric field sensing. The finite element method was used to analyze the conductor distribution, establish the coupling relationships among the electric field, transmission line, and measurement point, propose a sag inverse calculation model, and assess the impact of the transmission line parameter on the curved drooping measurement. Simultaneously, sag measurement schemes for single-round and dual-circuit lines were designed for multi-conductive lines, and measurement array studies were conducted. The vertical component of the electric field in space measured by the array was obtained, which could be used to perform conductor sag measurement simply and efficiently. The proposed method will facilitate the monitoring of the overhead transmission line status, which is conducive to the effective operation of the entire system.

## 1. Introduction

The stability of power systems is a key issue. As reliable power carriers, overhead transmission lines play an important role in long-distance power transmission and distribution. They connect the power supply to the distributed network and users. Therefore, real-time overhead transmission line status monitoring is important to maintain system stability and reliability [1,2].

Most transmission lines are exposed to the environment and operate in challenging working conditions. Harsh weather conditions threaten line security, thereby endangering the grid [3,4]. When lines are supported between two equal rod towers, sags tend to form. High temperatures during hot weather can cause transmission line extensions, thereby increasing sag. Sag reduces transmission line clearance, which increases the chances of forming short circuits with the surroundings. In severe cases, part of the transmission network may eventually crash, causing a power outage. Therefore, real-time sag measurements are important indicators of the operational health of transmission lines and are essential for the planning of prevention and treatment strategies.

The traditional sag measurement method generally involves contact, such as tension [5], inclination [6], and temperature [7,8] measurement methods, and needs to be connected directly with the conductor. During application, the tension measurement method should consider micro-sensitive factors, while the inclination measurement method is limited by the stiffness of the wire of the sensor installation position. Meanwhile, the temperature measurement method shows errors due to uneven temperature distribution along the line. In addition, the global positioning system [9,10] or a camera-based visual system [11] can be used to determine the position of the lines. Certain methods utilize an optomechanical system, which allows for the line’s sag change to be converted into a chirped fiber Bragg grating optical parameter change [12,13]. Other methods employ inspection robots on power lines to achieve remote control [14]. These devices are expensive and need to be installed on the electrical conductors of the transmission lines. Therefore, the performance of such devices may be affected by the electromagnetic interference of the conductor because the device must be positioned extremely close to the phase conductor.

Non-contact measurement equipment does not directly come into contact with the electrical object or affect system operation, nor does it provide portability and flexibility or enable high-frequency measurements [15]. The non-contact transmission system sag measurement method is, generally, based on electromagnetic sensing. The method involves measuring the electric or magnetic field by induction and then estimating the parameters, such as the gap from the conductor to the sensor and the sag. The magnetic field-based measurement scheme involves sensor coils or magnetic resistance sensors to perform magnetic field measurements [16,17], which is based on iteration fitting algorithms to estimate [18]. For example, three sensors were placed vertically on the tower for measurement purposes [19]; however, when the tower was tilted, previous technology was not convergent, as the proposed algorithm did not take into consideration the decreasing sag. In response, an algorithm based on the artificial immune system that accurately estimates the sag and tower tilt and detects the transmission system current was proposed [20]. Simultaneously, the magnetic field distribution was detected along the ground to avoid the trouble of installation on the tower. The electric-field-based measurement scheme mainly uses voltage monitoring and establishes the relationship between the voltage and the electrical field [21,22], and then allows for sag estimation through electric field measurements and anti-discrimination algorithms. The calculation method of the three-dimensional line voltage of the known structure of the three-dimensional model was proposed [23]. The application of voltage imbalance and sag measurement has been proposed to attain the amplitude and relative phase of the space potential of the transmission lines at two or more strategic positioning points in space [24]. A new type of overhead transmission line without contact sag and voltage monitoring methods has also been presented [25]. As long as a simple potential measurement array is required, it can be applied to dynamic conductor measurements. Most of these studies assume that the location of the transmission line is known and fixed, and it is necessary to determine the unknown sags and additional variables in the wire, which signifies that the number and locations of the sensors in the optimization process need to be further studied.

Evidently, the non-contact measurement method depends, to a large extent, on the accurate acquisition of the real-time parameters of the overhead transmission lines [24,26,27]. Transmission line currents change considerably with the load, which makes the measurement methods based on magnetic fields complicated, and electromagnetic distribution estimation using consistent iterative fitting algorithms is difficult. However, transmission line voltages are relatively constant and not easily affected by conditions, such as the surrounding environment and weather [28]. Therefore, the electric-field-based measurement method has received widespread attention.

To obtain stability and reliability, the sag measurement method discussed in this study is based on the sensation of the electric field, that is, the principle of calculating line sag by measuring the electric field. Most non-contact overhead transmission line measurement methods use simple two-dimensional equivalent effects [21,27]. In contrast, these methods lack the consideration of the actual conductor operation, including the effects of transmission line parameters on the sag measurement. In addition, the sag counter-discrimination calculation method described in the previous text [23,24,25] is complex and redundant, and the calculation itself is difficult. For the general situation, only the distribution status of three wires is considered, that is, a single-round line, and there is a lack of multiple-scene analysis.

In response to the aforementioned problems, this article emphasizes using the high flexibility and strong applicability of the finite element method and the characteristics of the actual state of simulating the conductor [29,30]. By analyzing the electric field of the ideal horizontal conductor and the actual catenary line conductor, the power field coupling relationship between the transmission line and measurement point was established. Combined with the simulation contrast, we can notice the difference between the ideal conductor and the actual conductor more intuitively. In order to make the sag calculation easier and more convenient, this article analyzes the approximate relationship of the sag and near-electric field through simulation and proposes an easy-to-operate sag inventory computing model. Additionally, the simulation demonstrated the effects of the transmission line parameters for sag measurement, and we analyzed the changing trends of the single-conductor sag with respect to the electric field, thereby verifying the feasibility of the sag measurement method. In response to the multi-conductive lines, a two-round line electric field analysis was conducted, the sag measurement schemes of single-and dual-round lines were designed, and measurement array studies were performed.

This article aims to verify the principles of the measurement method based on the electric field and highlights the issues that need attention when applying the measurement method in multiple scenarios. The main contributions of this article are as follows: (1) Combined with the finite element simulation, the study not only proposes a simple, convenient, and easy-to-operate sag counter-discrimination computing model but also analyzed the impact of the transmission line parameters on the sag measurement. This is conducive to proving the sag measurement proposed and verifying the feasibility of the method. (2) Multi-scenario distribution was considered so that the proposed measurement method is more extensive and can be applied to transmission lines of any structure. Further, this measurement method only requires the vertical electric field component and the basic structure of the lines measured by the horizontal array and can simply and efficiently implement conductive sag measurements to ensure a safe and reliable transmission line operation.

## 2. Sag Measurement Principle

### 2.1. Electrostatic Coupling Model between Transmission Line and Measuring Points

Before the inversion calculation and sag measurement, this study first established an electrostatic coupling model for forward calculation. The measured electric field value of the measuring point under the conductor was calculated from the parameters of the transmission line. We assume that the transmission line is suspended above the ground with a uniform load, and the conductor is simulated by a line charge located at its center.

#### 2.1.1. Catenary Line

**Figure 1:**
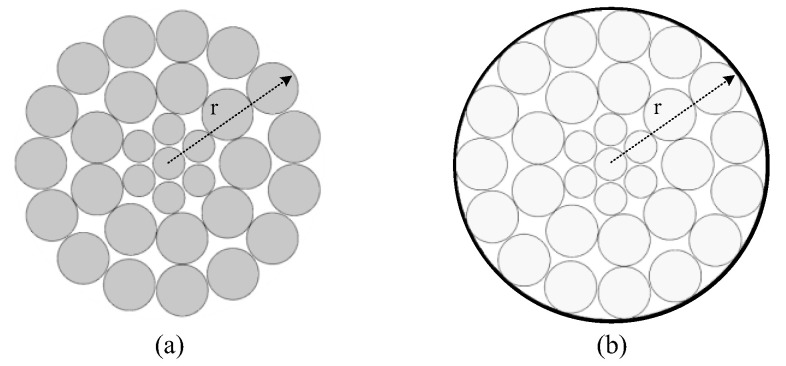
Schematic diagram of a single naked line, where r is the radius: (**a**) actual line; (**b**) simplified line.

Each conductor in the overhead transmission lines is composed of multiple stranded conductors (Figure 1a). To avoid numerical instability in space charge propagation caused by the detailed structure and modeling complexity, a simple circle of the conductor was adopted in the model (Figure 1b) [31].

For the bundle lines, the conductor radius can be calculated with the equivalent radius *r*, as shown in (1), where *n*_0_ is the number of conductor splits, *r*_0_ is the radius of sub conductor, and *R* is the geometric radius of sub conductor arrangement

(1) $$r=\sqrt[{n_{0}}]{r_{0}\times n_{0}\times R^{n_{0}-1}}.$$

**Figure 2:**
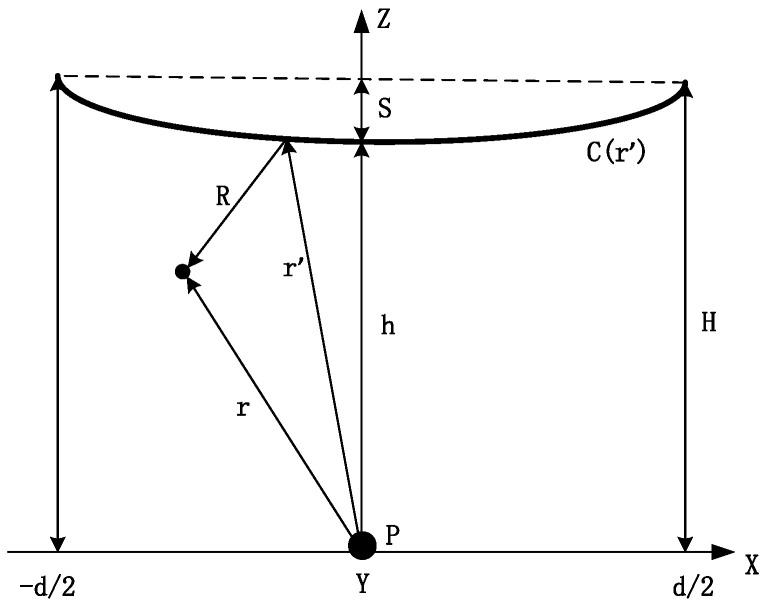
Line parameters of a single-conductor catenary. *H* is the maximum line height, *h* is the minimum height (*S* = *H* − *h*), and *d* is the line span length.

Generally, power transmission lines are erected in nearly periodic catenaries. Considering the details, the impact of the catenary on the electric field amplitude is evident. The effect of the weight on the line can be determined to obtain the basic contact mesh structure of a single-conductor catenary geometry (Figure 2) [22].

An approximate contact network equation was previously established, where the transmission line was located in the XZ plane and symmetrically distributed relative to the *Z* axis [32]. This relationship is given as

(2) $$z=S{\left(\frac{2x}{d}\right)}^{2}+h,$$

where *S* is the sag of the conductor, *d* is the span length, and *h* is the minimum height.

The electric field created by a single conductor catenary, placed in the air far from any other things can be obtained as follows:

(3) $$E\left(r\right)=\frac{1}{4\pi \varepsilon_{0}}\int_{C\left(r^{\prime }\right)}^{}\lambda \left(r^{\prime }\right)\frac{R}{M^{3}}dl^{\prime },$$

where *λ*(*r*’) is the wire charge density which depends on the voltage of the conductor. The integral is calculated on the curve *C*(*r*’), which defines the catenary, and *M = |R| = |r* − *r’|*, where *r* is the position of the measured point in space and *r*’ is the position of any point on the curve (Figure 2).

When the single conductor line is placed over a flat ground, an image line specularly symmetric to the air–ground interface plane has to be introduced to take into account the boundary conditions of the electric field and electric current in the conductive ground. The electric field over the ground is given by the superposition of the electric field generated by the actual catenary line *C*_1_(*r*’) and the image underground line *C*_2_(*r*’). The former expression then becomes:

(4) $$E\left(r\right)=\frac{1}{4\pi \varepsilon_{0}}\left[\int_{C_{1}\left(r^{\prime }\right)}^{}\frac{\lambda \left(r^{\prime }\right)R_{1}}{M_{1}{}^{3}}dl_{1}{}^{\prime }-\int_{C_{2}\left(r^{\prime }\right)}^{}\frac{\lambda \left(r^{\prime }\right)R_{2}}{M_{2}{}^{3}}dl_{2}{}^{\prime }\right],$$

(5) $$M_{1}=\left|R_{1}\right|,R_{1}=\left(x-x^{\prime }\right)\vec{X}+\left(y-{y^{\prime }}_{0}\right)\vec{Y}+\left(z-z^{\prime }\right)\vec{Z},$$

(6) $$M_{2}=\left|R_{2}\right|,R_{2}=\left(x-x^{\prime }\right)\vec{X}+\left(y-{y^{\prime }}_{0}\right)\vec{Y}+\left(z+z^{\prime }\right)\vec{Z},$$

where (*x*, *y*, *z*) are the spatial coordinates of the point to be tested on the ground and (*x*’, *y*’, *z*’) are the coordinates of any point of the wire. *y*’_0_ is the constant position of the single-direction line on the Y axis. Here, *y*’_0_ = 0.

It is enough to use the voltage of the single conductor line to obtain $\lambda \left(r^{\prime }\right)$:

(7) $$V\left(r\right)=\frac{1}{4\pi \varepsilon_{0}}\left[\int_{C_{1}\left(r^{\prime }\right)}^{}\frac{\lambda \left(r^{\prime }\right)}{M_{1}}dl_{1}{}^{\prime }-\int_{C_{2}\left(r^{\prime }\right)}^{}\frac{\lambda \left(r^{\prime }\right)}{M_{2}}dl_{2}{}^{\prime }\right].$$

The measurement point discussed in this article is considered to be in the middle of the conductor, and the line span length is much greater than the minimum line height (d≫h). This point is very far from the pole tower, so the impact of the tower can be ignored. So, we assume for this calculation that the charge is constant along the line. That is, $\lambda \left(r^{\prime }\right)$ is a constant when $V=V_{0}$.

The electric field generated by the single conductor at P (Figure 2) is vertical and expressed as:

(8) $$E_{0}=\frac{\lambda \left(r^{\prime }\right)}{2\pi \varepsilon_{0}}\int_{C\left(r^{\prime }\right)}^{}\frac{z^{\prime }}{M_{0}{}^{3}}dl^{\prime },\;$$

(9) $$M_{0}=\left|R_{0}\right|,R_{0}=z^{\prime }\vec{Z}.$$

In summary, the electric field value is related to the contact network equation. In combination with Equation (2), the electric field at P is expressed as:

(10) $$E_{0}=\frac{\lambda \left(r^{\prime }\right)}{2\pi \varepsilon_{0}}\int_{-\frac{d}{2}}^{\frac{d}{2}}\frac{1}{{\left(S{\left(\frac{2x}{d}\right)}^{2}+h\;\right)}^{2}}dx.\;$$

#### 2.1.2. Horizontal Line

The common practice is to assume that power line conductors are straight horizontal lines and parallel to the flat ground. The “average” height is taken between the maximum and minimum heights of the line, ignoring the sag caused by the line weight [33]. To simplify this problem, the distance from the lowest point of the conductor to the ground was selected as an effective height in this article.

**Figure 3:**
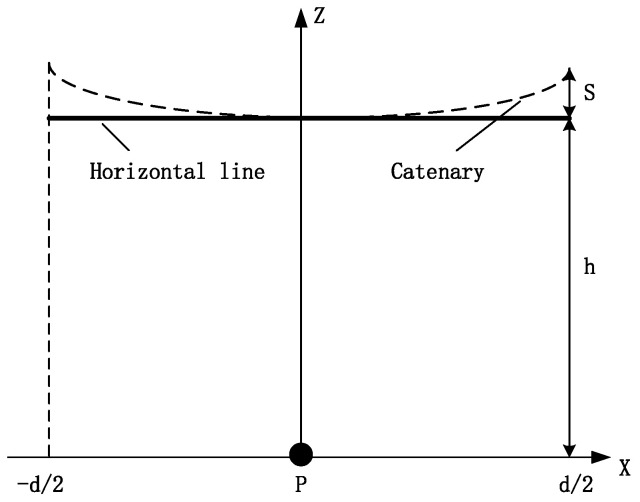
Parameters of a horizontal line.

To perform visual comparisons, an ideal horizontal line was produced by drawing the surface electric field generated by the minimum horizontal line (*h* = *H* − *S*) (Figure 3).

Applying the same method used for a catenary single conductor line, we can calculate the electric field created by a horizontal single conductor line located at a height above the ground:

(11) $$V\left(r\right)=\frac{\lambda_{0}}{2\pi \varepsilon_{0}}\ln\left(\frac{M_{2}}{M_{1}}\right).$$

(12) $$M_{1}=\left|R_{1}\right|,R_{1}=\left(y-{y^{\prime }}_{0}\right)\vec{Y}+\left(z-z^{\prime }\right)\vec{Z},$$

(13) $$M_{2}=\left|R_{2}\right|,R_{2}=\left(y-{y^{\prime }}_{0}\right)\vec{Y}+\left(z+z^{\prime }\right)\vec{Z}.$$

We can obtain:

(14) $$\lambda_{0}=\frac{2\pi \varepsilon_{0}V_{0}}{\ln\frac{h}{r}}.$$

The field created by the horizontal conductor lines is:

(15) $$E\left(r\right)=\frac{\lambda_{0}}{4\pi \varepsilon_{0}}\left(\frac{R_{1}}{M_{1}{}^{2}}-\frac{R_{2}}{M_{2}{}^{2}}\right).$$

Therefore, the electric field at point P was generated by a horizontal line on the ground, which can be expressed as:

(16) $$E_{0}=\frac{\lambda_{0}}{2\pi \varepsilon_{0}h}\left(\frac{d}{\sqrt{h^{2}+{\left(\frac{d}{2}\right)}^{2}}}\right).$$

Based on the numerical analysis, we can obtain the accurate calculation of the electric field generated by a single power transmission line. However, electric fields of transmission lines can be obtained through other methods, such as finite elements, limited differentials, or charge simulation. Here, the mathematical treatment of the finite element method is more convenient and we only need to obtain an approximate solution to compare the electric field created by a single catenary with that created by a single horizontal line.

**Figure 4:**
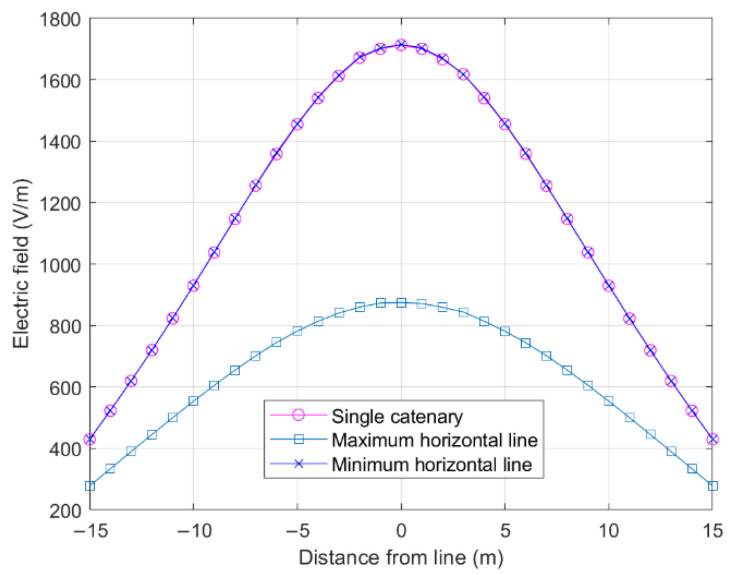
Comparison of the electric fields of horizontal and catenary lines.

Assuming that the parameters of the single line were the same, the catenary, maximum horizontal line, and minimum horizontal line were simulated and the electric field values generated by them at P were calculated. Figure 4 depicts the differences between the electric fields. It can be seen that the electric fields at P generated by a single catenary and the minimum horizontal line are very similar.

### 2.2. Inverse Calculation of Sag

#### 2.2.1. Theory

The essence of the sag inverse calculation was based on the distribution of the electric field of the line to establish the relationship between the conductor sag and electric field distribution. The sag was achieved by collecting electric field data generated on the ground during the transmission line operation.

From Equations (10) and (16), it can be seen that there is a monotonous incremental relationship between the electric field value and sag of the power transmission lines. Correspondingly, the sags can be deduced in reverse by measuring the electric field value, as follows (where *F* represents the functional relationship between *E* and *S*, and *F*^−1^ represents the counter-function):

(17) $$E_{0}=F\left(S\right)\Rightarrow S=F^{-1}\left(E_{0}\right).$$

According to numerical analysis, the measured value *E* contains the information of the single conductor parameters and the measured point position. When all conductor parameters (*H*, *d*, *U*) and the point position (*x*, *y*, *z*) are known, the conductive sag is calculated by measuring the electric field value.

For a single line, the measured point discussed in this article was selected under the middle of the conductor: x=0; y=0; z=0.

#### 2.2.2. Simplified Computing Model

**Figure 5:**
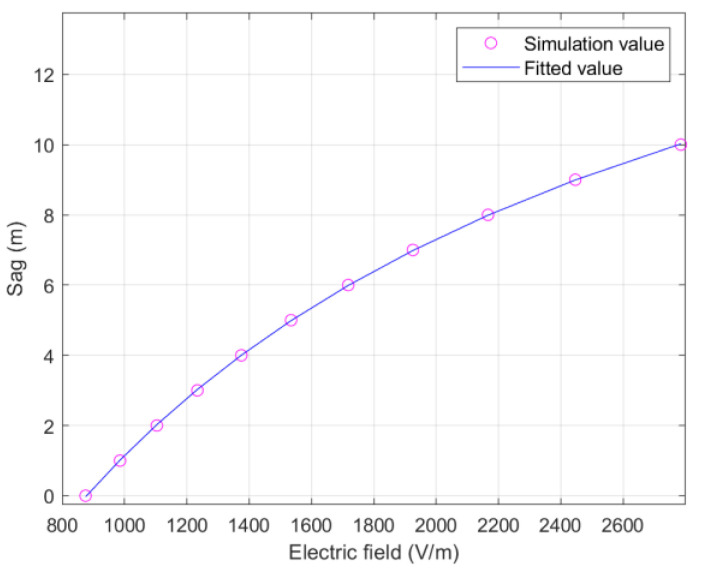
Relationship between sag and electric field.

Although there is a monotonous relationship between the conductive sag with the electric field, it is non-linear. In order to make sag measurement more convenient and easier to operate, the similar relationship between the sag and electric field can be further simplified to the calculation function without considering too much accuracy. Due to multiple large-scale operations and geometric conductor coupling state simulations, we used the finite element method for this purpose in the article. A similar functional relationship between the sag and electric field was obtained by calculating the simulation electric field values corresponding to the sag and then fitting the simulation curve (Figure 5). In application, the sag of transmission lines were estimated by the measured electric field value.

Here, the span length *d* was 300 m, tower height *H* was 20 m, and wire voltage was 110 kV. The functional relationship corresponding to the fit curve is:

(18) $$S=\frac{a\times E_{0}+b}{E_{0}+c}=a+\frac{b-ac}{E_{0}+c}\;,$$

where *S* is the sag and *E*_0_ is the electric field at P; moreover, *a* = 21.31, *b* = −1.871 × 10^4^, and *c* = 1270.

Through an analysis of the electric field, it was determined that the wire charge density *λ*(*r*′) in the formula depended on the voltage of the conductor. That is, the power field strength was proportional to the wire voltage. On this basis, the density of the line charge was calculated first. The wire charging density of the unit conductor voltage was *λ*_0_(*r*′). Subsequently, Equation (18) becomes:

(19) $$y=\frac{Ax+B}{x+C}=A+\frac{B-AC}{x+C}\;,$$

where *y* represents the sag and *x* represents the electric field under the unit conductor voltage.

The sag is not only related to the electric field value but is also affected by coefficients *A*, *B*, and *C*. Among them, *A* is the most influential. *A*, *B*, and *C* are related to the configuration parameters of the transmission line. If the line configuration remains unchanged, the stronger the electric field and the greater the sag. Therefore, the sag measurement of the transmission line is also affected by the different degrees of line parameters.

## 3. Effects of Line Parameters on Sag Measurement

### 3.1. Span Length

**Figure 6:**
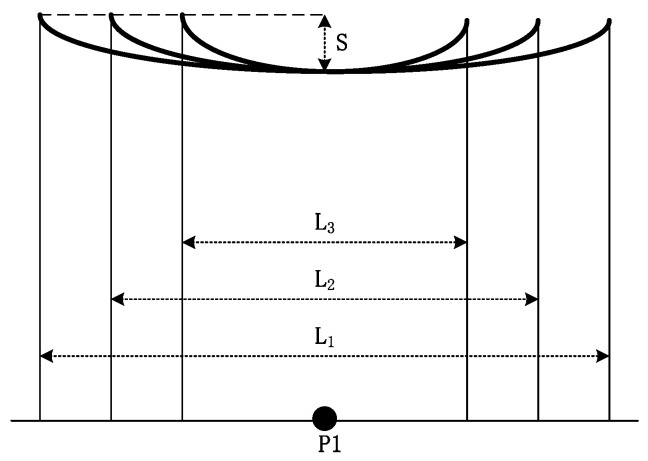
Span configurations of different lengths.

Figure 6 depicts the case in which changes in the sag of lines with span are not considered. Here, it is assumed that the sag of the lines does not change as the span length increase (L_1_ > L_2_ > L_3_, *S*_1_ = *S*_2_ = *S*_3_ = *S*).

**Figure 7:**
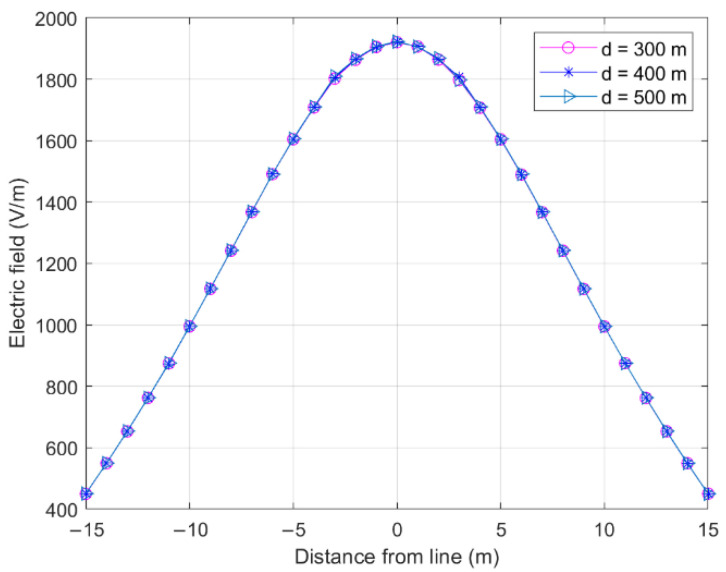
Effect of span length on cross-level electric field (*S*_1_ = *S*_2_ = *S*_3_).

Figure 7 shows the influence of the span length on the electric field on both sides of the longitudinal distance wire at P1 in Figure 6.

A smaller longitudinal distance from the wire would result in a greater electric field value. The influence of the span length on the cross-central electric field was extremely low, and the electric fields generated by the wires of different lengths were approximately identical, as the wire length was much greater than the distance from the measurement point to the wire, resulting in the wires contributing less to the electric field.

**Figure 8:**
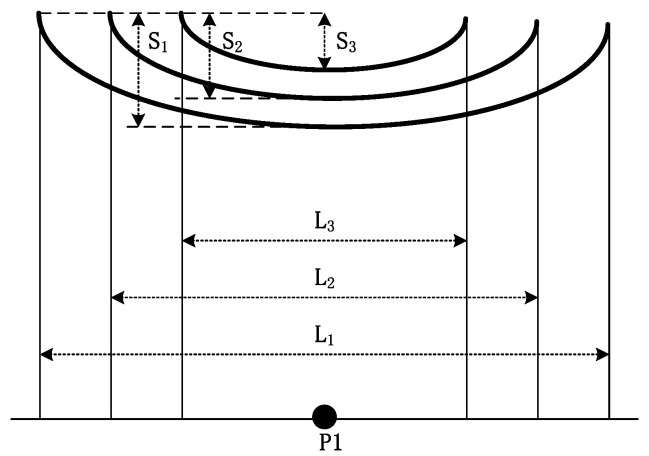
Slip configurations of different lengths and sags.

The effect of the research span length on the calculated electric field can be obtained by considering the change in span (Figure 8).

**Figure 9:**
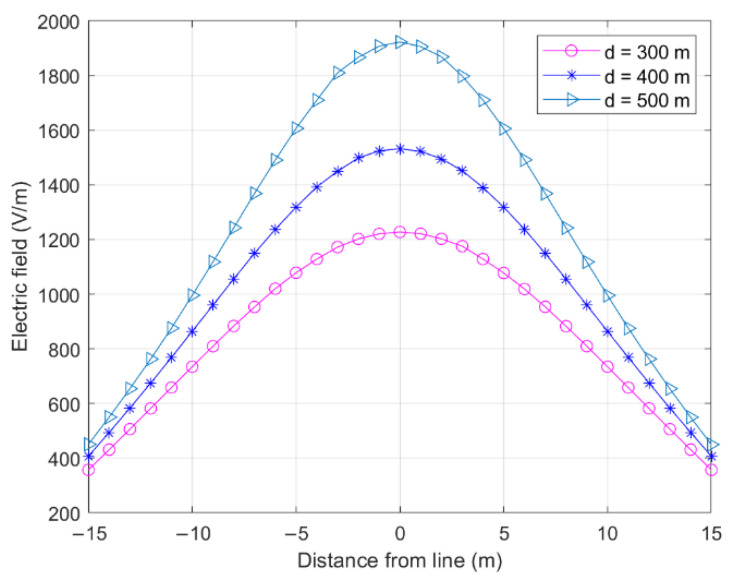
Effect of span length on cross-level electric field (*S*_1_ > *S*_2_ > *S*_3_).

Let us assume that the sag decreases with decreasing span (L_1_ > L_2_ > L_3_, *S*_1_ > *S*_2_ > *S*_3_) [34]. Subsequently, the span length at P1 in the electric field on both sides would be affected when the sag of the wire changes with the span distance (Figure 9).

In this case, the span length has a greater effect on the cross-medium-down electric field. In addition, the electric fields generated by the wires of different lengths are significantly different. A larger span length would result in a greater peak electric field being generated by the wire. Therefore, the effect of the sag is greater than that of the span (Figure 8).

**Figure 10:**
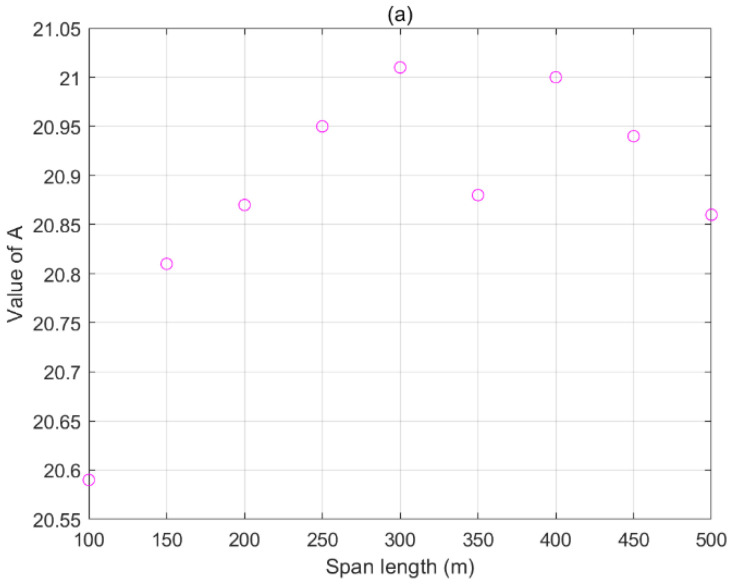

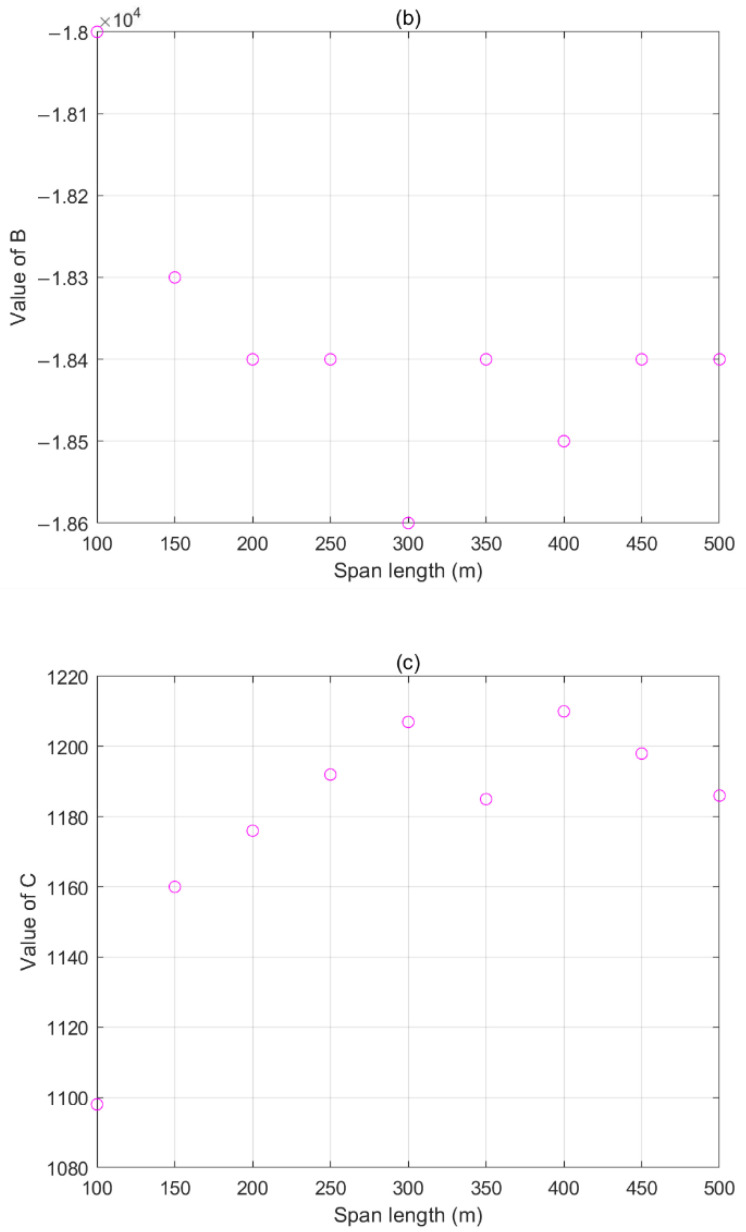
Effects of span length on coefficients: (**a**) *A* of different span lengths; (**b**) *B* of different span lengths; (**c**) *C* of different span lengths.

The effect of the span length on the sag measurement is mainly reflected in *A*, *B*, and *C*. Assuming that the height of the tower remained unchanged (*H* = 20 m), the simulated spans of different lengths and electric fields under different sags were calculated to obtain the coefficient values and analyze the relationships between the span length and coefficients. The simulation sizes of *A*, *B*, and *C* corresponding to different span lengths are shown in Figure 10.

When the line was transformed in the range of 100–500 m, the maximum change rates of *A*, *B*, and *C* were approximately 2.04%, 3.33%, and 10.2%, respectively. *A*, *B*, and *C* were less affected by the span length, their values were slightly changed, and each coefficient had no evident functional relationship with the span growth.

Therefore, when the electric field value was fixed, the wire length increased, and the corresponding sag changes were small. Thus, we can regard the sag as remaining unchanged. This simulation result is in agreement with the theoretical analysis.

### 3.2. Tower Height

**Figure 11:**
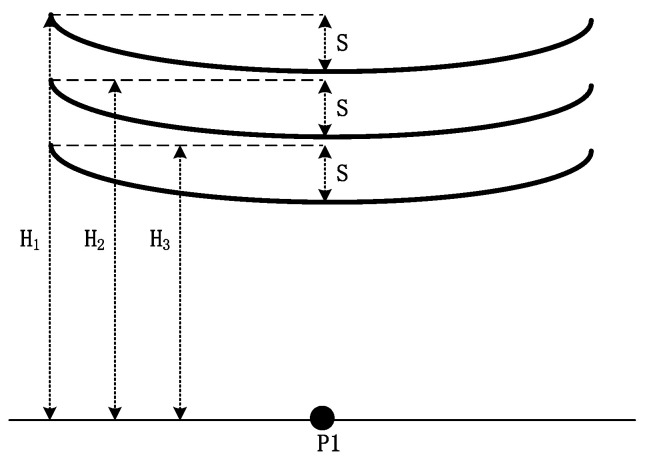
Tower configurations of different heights.

If a single-guide line is reduced with the tower height, the wire sag remains unchanged (*H*_1_ > *H*_2_ > *H*_3_) (Figure 11).

**Figure 12:**
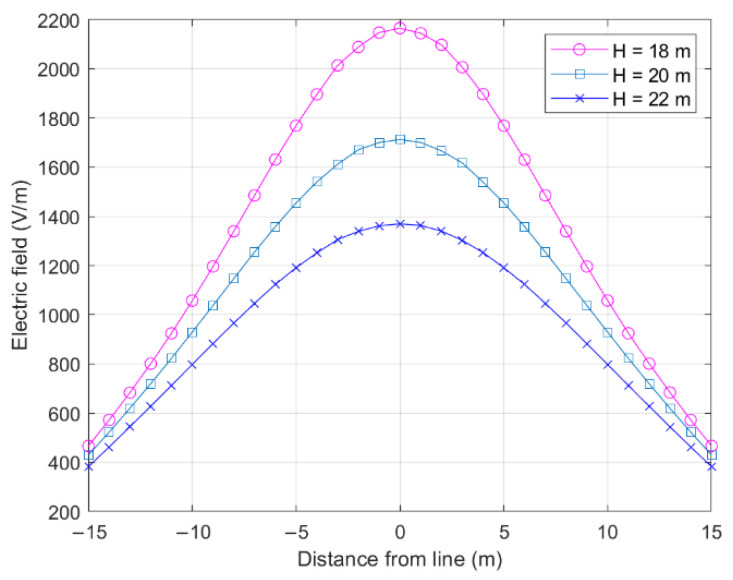
Effect of tower height on cross-level electric field.

The effect of the height of a single-rooted conductor tower (maximum line height) on the electric field is shown in Figure 12.

A lower tower height would result in a stronger nearby field. This relationship is due to the decreases in the distance and the maximum height of the line, which reduces the gap between the line and P1.

**Figure 13:**
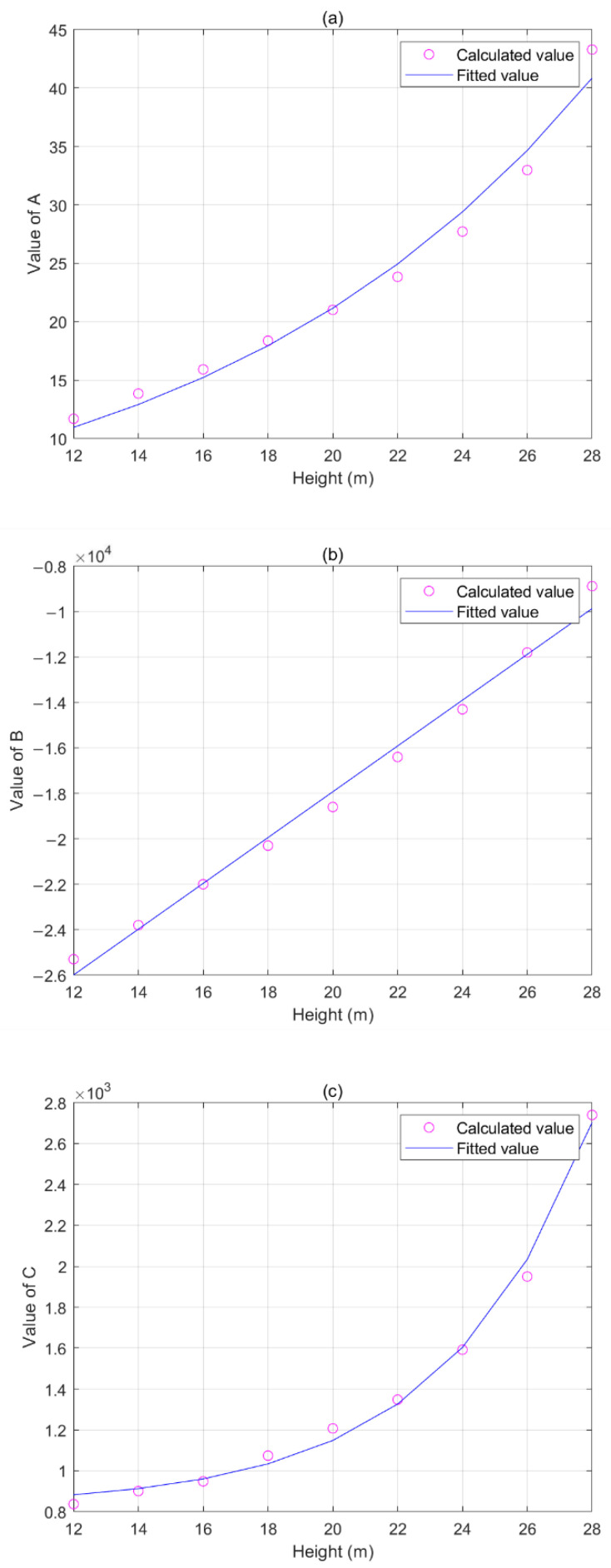
Effects of tower height on coefficients: (**a**) *A* of different tower heights; (**b**) *B* of different tower heights; (**c**) *C* of different tower heights.

To study the effects of the tower height on *A*, *B*, and *C*, it was assumed that the span lengths of the wires were unchanged (*d* = 300 m). Each parameter value can be obtained by calculating the electrical fields at different towers to analyze the relationships between the tower height and parameters. The simulation sizes of *A*, *B*, and *C* corresponding to different tower heights are shown in Figure 13.

*A*, *B*, and *C* gradually increased as the tower height increased from 12 to 28 m. The fit curves were obtained through the approximate functional relationships between the fitting coefficients and tower height (Figure 13). For *A*, *B*, and *C*, these relationships can be respectively expressed as follows:

(20) $$A\left(H\right)=4.081\times e^{0.08227H},$$

(21) $$B\left(H\right)=1008\times H-3.809\times 10^{4},$$

(22) $$C\left(H\right)=3.874\times e^{0.2208H}+827.5,\;$$

where *H* is the tower height, that is, the maximum line height.

The tower height had the most influence on *A* and *B*, which increased as the tower height increased. The following conclusion was obtained according to the principle of sag measurement: the tower height increased and the corresponding sag became larger when the electric field value was unchanged. This simulation result is in agreement with the theoretical analysis.

### 3.3. Tower Height Difference

**Figure 14:**
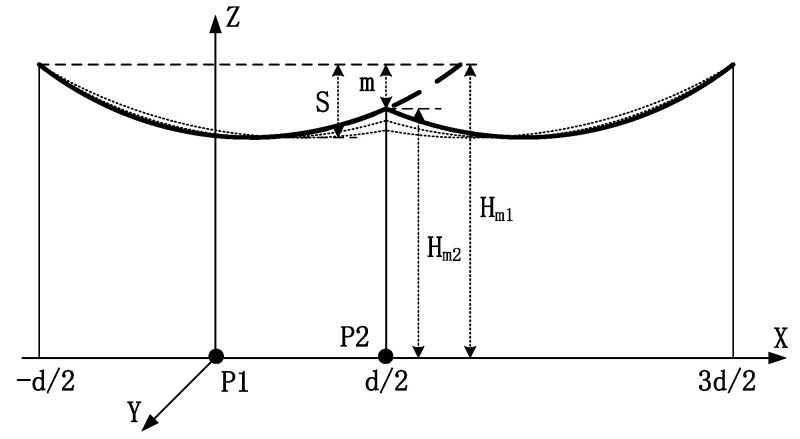
Schematic diagram of transfer cable configuration.

The transfer cable configuration schematic diagram is shown in Figure 14.

The lowest point of the catenary was located in the cross when considering the neighboring tower height in the transmission line under normal circumstances. In fact, the tower heights of multiple lines were different. There was a height difference with respect to the adjacent tower, m (m = *H*_m2_ − *H*_m1_) for a single-conductor catenary line (Figure 14). At this time, the lowest point of the catenary line deviated from the cross. As the height difference of the tower changed, it was assumed that the verticality of the wires remained unchanged.

**Figure 15:**
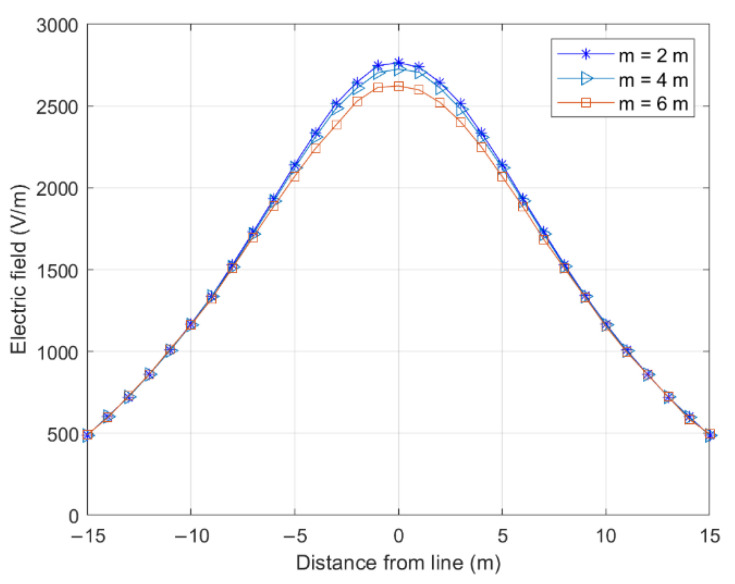
Effect of height difference on cross-level electric field.

**Figure 16:**
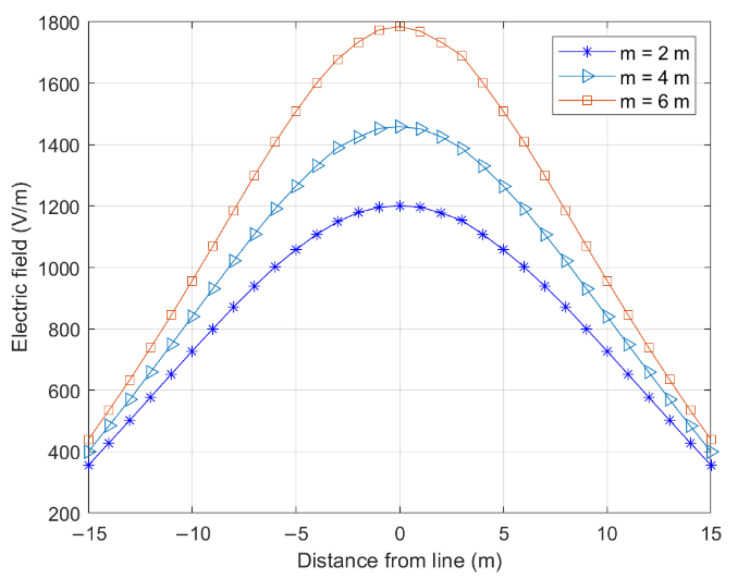
Effect of height difference on electric field of tower position.

The effects of the tower height on different tower positions (P1 and P2 in Figure 14) and the electric field on both sides of the longitudinal wires are shown in Figure 15 and Figure 16.

With an increasing tower height difference, the electric field at P1 gradually decreased, and that at P2 gradually increased. These changes occurred because the lowest point of the catenary shifted to the right as the height difference increased, gradually deviating from the cross-center position. This characteristic decreased the electric field at P1. Simultaneously, the gap between the line and P2 on the ground decreased, increasing the electric field on the ground. Notably, the tower height difference had a greater effect on the electric field of the tower position than the cross-center position.

**Figure 17:**
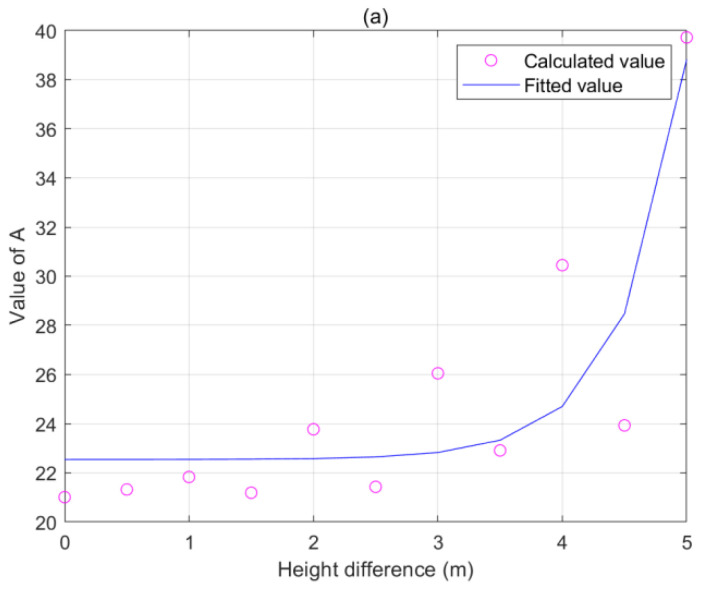

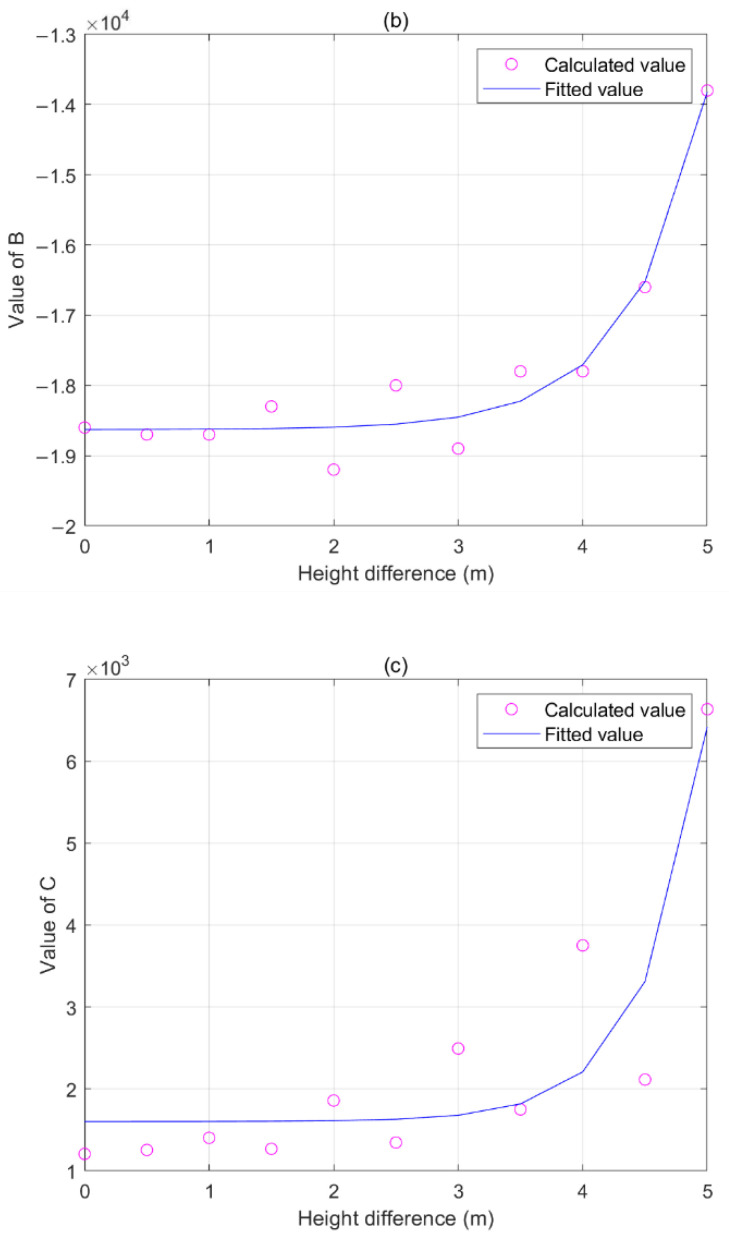
Effects of tower height difference on coefficients: (**a**) *A* of different height differences; (**b**) *B* of different height differences; (**c**) *C* of different height differences.

The effect of the tower height difference on the sag measurement was explored, assuming that the span length and maximum tower height were unchanged. The relationships between the tower height difference and parameters were further analyzed by simulating the electric field at P1 at different tower heights and calculating *A*, *B*, and *C*. The simulation parameters corresponding to different tower heights are presented in Figure 17.

When the height difference between the towers increased from 0 to 5 m, the volatility of *A*, *B*, and *C* increased. There were approximate functional relationships between the coefficients and the tower height difference. The fit curves could be obtained by simulating the approximate functions, as shown in Figure 17:

(23) $$A\left(m\right)=6.686\times 10^{-4}\times e^{2.02m}+22.54,$$

(24) $$B\left(m\right)=1.256\times e^{1.65m}-1.863\times 10^{4},$$

(25) $$C\left(m\right)=0.1526\times e^{2.072m}+1602,$$

where m is the height difference of the towers.

According to the sag measurement principle and Equation (19), the effect of *A* on the sag was greater than that of *B* and *C*. Therefore, the greater the tower height difference, the larger the corresponding sag when the electric field value was fixed. This simulation result is basically in agreement with the theoretical analysis.

This article analyzes the effects of the line parameters on sag measurement based on the finite element simulation, which directly reflects the change of line parameters on the coefficients in Equation (19). It can be concluded that the impact of the tower height on the sag measurements was greater than the impact of the tower height differences on the sag measurements, which was in turn greater than the effect of the span length. It is worth noting that by analyzing the changing trend of the line parameters of the equation coefficients, we can verify the methods proposed. In other words, the principle of measuring sags using electric field countermeasures is feasible.

## 4. Multi-Conductor Sag Measurement

### 4.1. Arrangement

**Figure 18:**
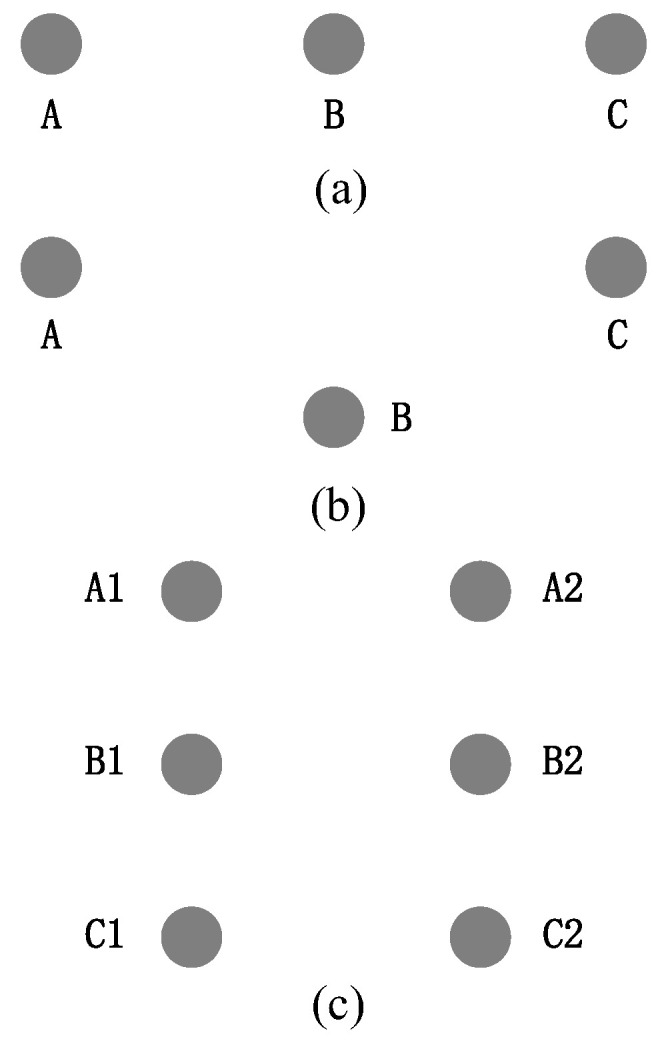
Line arrangement methods: (**a**) horizontal arrangement; (**b**) triangular arrangement; (**c**) double-back vertical arrangement.

There are three main distribution methods for high-voltage overhead lines: horizontal, triangular, and double-back vertical arrangements (Figure 18). Single-round lines are shown in Figure 18a,b, and a dual-round line of the same tower is shown in Figure 18c.

In the case of multiple conductors, the induction charge on each conductor was calculated using the potential coefficient [34]. For the three contour points on the surface of the equivalent conductor, the potential was equal to the surface voltage of the conductor.

**Figure 19:**
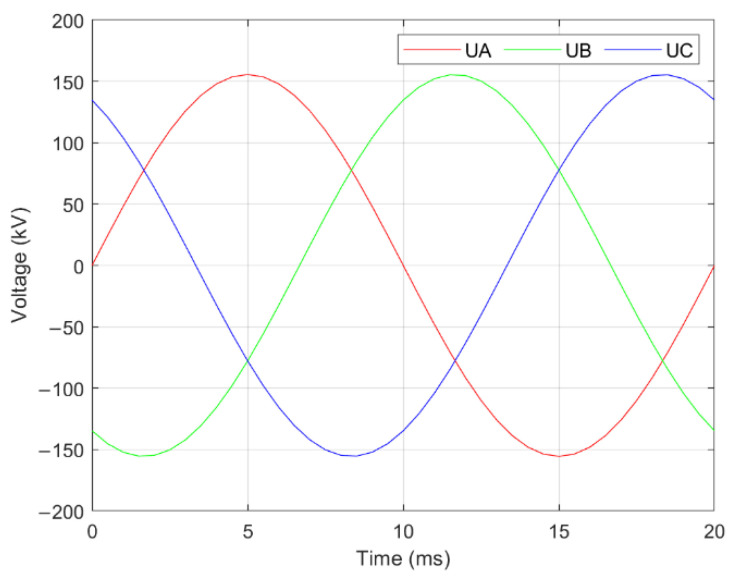
Wire voltage changes over time.

Under normal circumstances, the voltage amplitude of each phase of the transmission line was equal, and the corner lagged by 120°. Therefore, the wire voltage changed periodically (Figure 19).

The catenary line is equivalent to an infinitely long conductor line for simplicity [35]. Its height is from the lowest point to the ground, as shown in the horizontal straight wire in Figure 3. Therefore, the two-dimensional section of this point was chosen for calculation. Further, only the effective value of the voltage amplitude was considered in this article, because the voltage maximum amplitude was consistent for each conductor; hence, the linear charge density of the conductors could be considered the same to simplify the calculations.

**Figure 20:**
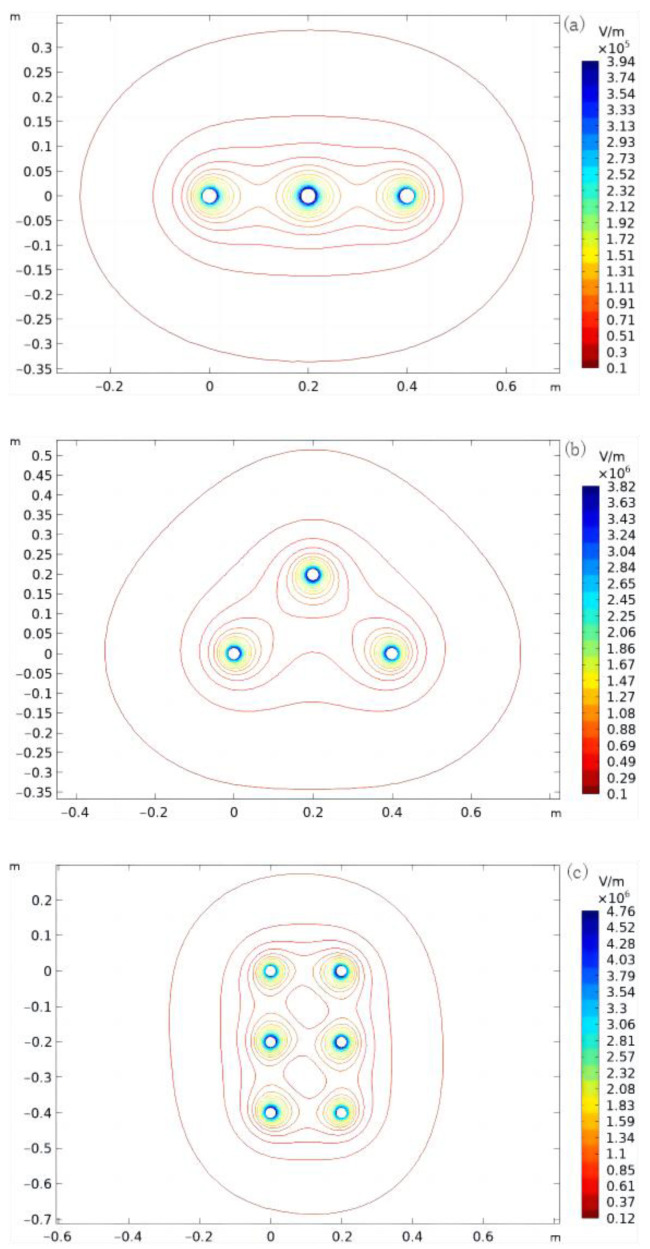
Arrangements of three-phase wires with different distributions: (**a**) horizontal; (**b**) triangular; (**c**) dual-round.

Figure 20 shows three-phase wires with different arrangements. According to the simulation, the multi-conductor transmission wire electric fields were coupled with each other. The electric field on the ground was affected by the superposition effect of all conductors.

In this case, the electric field generated by the single-conductor catenary on the ground is based on the calculated electric field of an unlimited horizontal straight line:

(26) $$E_{i}=\frac{\lambda }{2\pi \varepsilon_{0}R_{i}}\;,\;\;\lambda =\frac{2\pi \varepsilon_{0}U_{e}}{\ln\frac{h}{r}}\;,$$

where *U_e_* is the effective amplitude of the wire voltage, *r* is an equivalent conductor radius, and *h* is the distance from the lowest point to the ground. *R_i_* is the distance from the center point of the equivalent guide contour on the ground.

According to superposition technology, the electric field generated by the multi-conductor suspension lines on the ground is given as:

(27) $$E=\sum_{i=1}^{n}E_{i},$$

where *n* represents the number of conductors.

When using an unlimited long model for inverted sag calculations, the unknown variable needs to be obtained according to the number of conductors to determine the number of measurement points and their locations.

### 4.2. Single-Round Lines

When the voltages and guide positions are determined for single-round lines, the sag calculation contains one unknown variable if the verticality of each phase wire is consistent, and three if the verticality is considered inconsistent. The sensors can be placed parallel to different positions under the horizontal ground so that a phase conductor can measure the space field, as shown in Figure 19 and Figure 20. Three measurement points form a measurement array. Therefore, three equations can be obtained to find the sag of the solution to the return line.

The direction of detection is generally parallel or vertical with respect to the main axis direction of the sensor because the current existing electric field sensors can only detect one- or two-dimensional power field intensities. Therefore, the vertical component of the electric field was measured in this study for simplicity and convenience.

**Figure 21:**
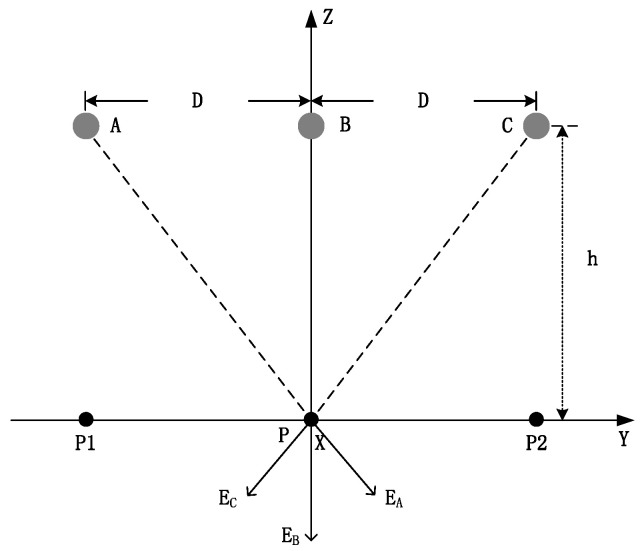
Horizontal arrangement of a three-phase line.

*D* is the horizontal distance between the wires of each phase, and *h* is the distance from the lowest point to the ground (Figure 21). The electric fields generated on the ground are *E_A_*, *E_B_*, and *E_C_*.

If the height of the equivalent conductor to the ground is constant, then *h* = *H* − *S*. The electric field at P can be expressed as:

(28) $$E=\sum_{i=1}^{3}E_{i}=\sum_{i=1}^{3}\frac{\lambda }{2\pi \varepsilon_{0}R_{i}},$$

(29) $$R_{B}=h,R_{A}=R_{C}=\sqrt{h^{2}+D^{2}}.$$

According to symmetry, *E_A_* = *E_C_*_,_ and the electric field at P is the vertical component of the electric field:

(30) $$E=E_{B}+2E_{A}\frac{h}{\sqrt{h^{2}+D^{2}}}=\frac{\lambda }{2\pi \varepsilon_{0}}\left(\frac{1}{h}+\frac{2h}{h^{2}+D^{2}}\right).\;$$

If the verticality of the three-phase wire is not equal, the distances from the lowest points to the ground are *h_A_*, *h_B_*, and *h_C_*_,_ where *h_A_* = *H* − *S_A_*, *h_B_* = *H* − *S_B_*, and *h_C_* = *H* − *S_C_*. Here, the vertical components of the electric field at P are:

(31) $$R_{A}=\sqrt{h_{A}{}^{2}+D^{2}},R_{B}=h_{B},R_{C}=\sqrt{h_{C}{}^{2}+D^{2}},$$

(32) $$\cos\alpha =\frac{h_{A}}{\sqrt{h_{A}{}^{2}+D^{2}}},\cos\beta =\frac{h_{C}}{\sqrt{h_{C}{}^{2}+D^{2}}}\;,$$

(33) $$E=E_{A}\cos\alpha +E_{B}+E_{C}\cos\beta =\frac{\lambda }{2\pi \varepsilon_{0}}\left(\frac{h_{A}}{h_{A}{}^{2}+D^{2}}+\frac{1}{h_{B}}+\frac{h_{C}}{h_{C}{}^{2}+D^{2}}\right).$$

Similarly, the vertical components of the electric fields at P1 and P2 are, respectively:

(34) $$E_{1}=\frac{\lambda }{2\pi \varepsilon_{0}}\left(\frac{1}{h_{A}}+\frac{h_{B}}{h_{B}{}^{2}+D^{2}}+\frac{h_{C}}{h_{C}{}^{2}+{\left(2D\right)}^{2}}\right),\;$$

(35) $$E_{2}=\frac{\lambda }{2\pi \varepsilon_{0}}\left(\frac{1}{h_{C}}+\frac{h_{B}}{h_{B}{}^{2}+D^{2}}+\frac{h_{A}}{h_{A}{}^{2}+{\left(2D\right)}^{2}}\right).$$

**Figure 22:**
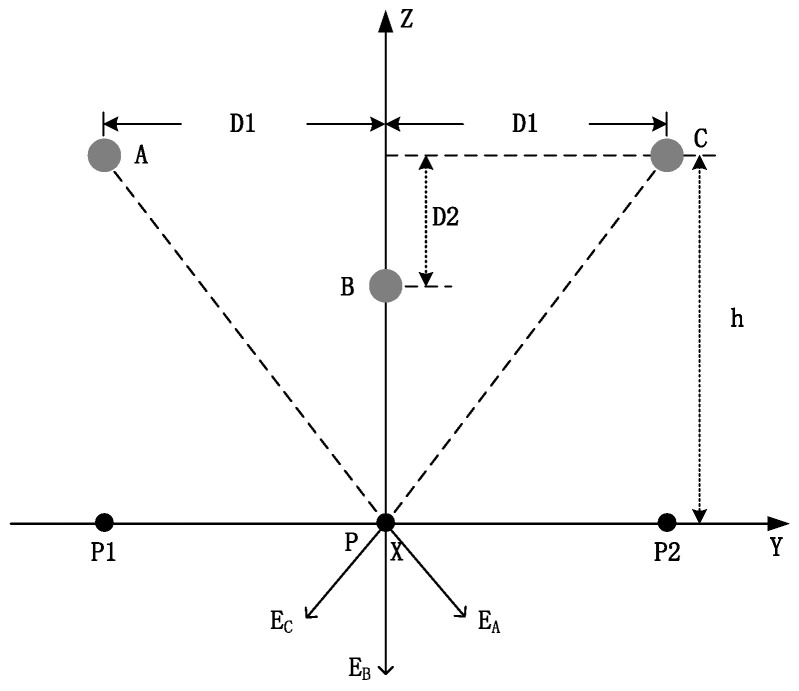
Triangular arrangement of a three-phase wire.

In Figure 22, *D*1 and *D*2 are the horizontal and vertical distances between the wires, respectively. The electric fields generated on the ground are *E_A_*, *E_B_*, and *E_C_*.

The same method is used for the horizontal arrangement. If the verticality of the three-phase wire is equal, the electric field at P is given as:

(36) $$R_{B}=h-D2,R_{A}=R_{C}=\sqrt{h^{2}+D1^{2}},$$

(37) $$E=\frac{\lambda }{2\pi \varepsilon_{0}}\left(\frac{1}{h-D2}+\frac{2h}{h^{2}+D1^{2}}\right).$$

If the verticality of the three-phase wire is not equal, the vertical components of the electric fields at P1 and P2 are:

(38) $$E=\frac{\lambda }{2\pi \varepsilon_{0}}\left(\frac{h_{A}}{h_{A}{}^{2}+D1^{2}}+\frac{1}{h_{B}}+\frac{h_{C}}{h_{C}{}^{2}+D1^{2}}\right),$$

(39) $$E_{1}=\frac{\lambda }{2\pi \varepsilon_{0}}\left(\frac{1}{h_{A}}+\frac{h_{B}}{h_{B}{}^{2}+D1^{2}}+\frac{h_{C}}{h_{C}{}^{2}+{\left(2D1\right)}^{2}}\right),$$

(40) $$E_{2}=\frac{\lambda }{2\pi \varepsilon_{0}}\left(\frac{1}{h_{C}}+\frac{h_{B}}{h_{B}{}^{2}+D1^{2}}+\frac{h_{A}}{h_{A}{}^{2}+{\left(2D1\right)}^{2}}\right),$$

where *E_n_* is the vertical component of the electric field measured at the *n*th metering point. *F_n_* is the measurement matrix between the nth metering point and vertical values of each phase. After obtaining this information and other data, such as the voltage, the sag can be calculated by measuring the electric field component. Therefore, the measurement array can be set up, and the counter-exploring calculation of the three-phase line sag can be achieved. The connection between sag and electric field can be expressed as:

(41) $$\left(\begin{array}{c} S_{A}\\ S_{B}\\ S_{C} \end{array}\right)=\left(\begin{array}{ccc} a_{11} & a_{12} & a_{13}\\ a_{21} & a_{22} & a_{23}\\ a_{31} & a_{32} & a_{33} \end{array}\right)\left(\begin{array}{c} E_{1}\\ E_{2}\\ E_{3} \end{array}\right)=F_{n}\left(\begin{array}{c} E_{1}\\ E_{2}\\ E_{3} \end{array}\right),$$

where *n* = 3.

### 4.3. Dual-Round Lines

When the voltages and conductors are determined for dual-round lines, the sag calculation contains one unknown variable if the vertical degree of each phase wire is consistent and six unknown variables if the verticality is considered inconsistent. The sensor can be placed in parallel at different positions on the horizontal ground under the conductor to measure the spatial field (Figure 21). The three measurement points form a measurement unit, and the two measurement units form a measurement array. Thus, we can obtain six equations to solve the sag of the dual-round line.

**Figure 23:**
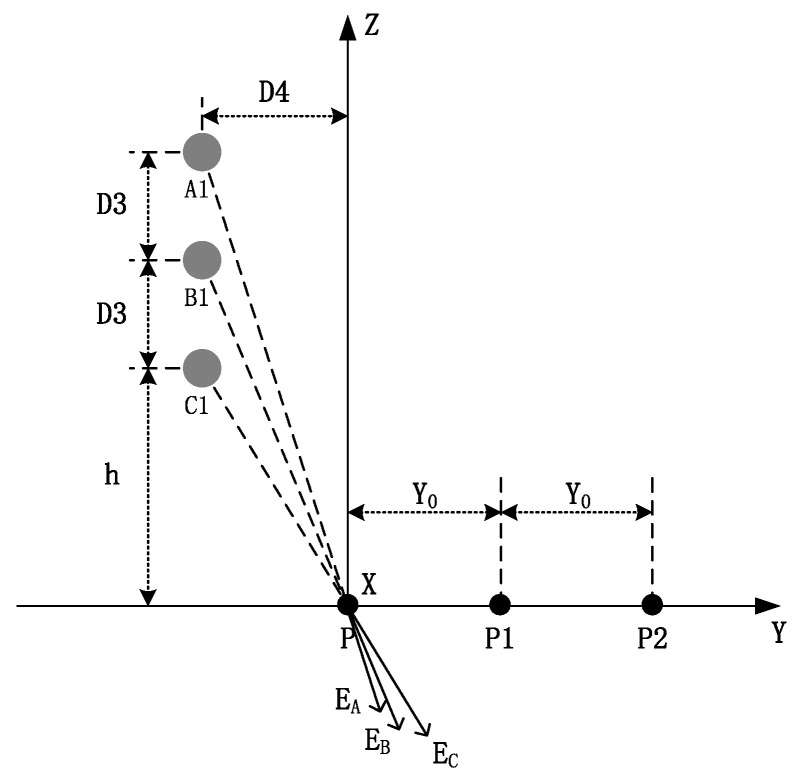
Vertical arrangement of a three-phase wire.

Figure 23 shows a unilateral vertically arranged three-phase wire, where *D*3 is the vertical distance between the wires.

The electric fields generated on the ground are *E_A_*, *E_B_*, and *E_C_*. Similar to the single-round line measurement method, the expression of the electric field at P is shown below if the verticality of the three-phase wire is equal:

(42) $$E=\frac{\lambda }{2\pi \varepsilon_{0}}\left(\frac{h}{h^{2}+D4^{2}}+\frac{h+D3}{{\left(h+D3\right)}^{2}+D4^{2}}+\frac{h+2D3}{{\left(h+2D3\right)}^{2}+D4^{2}}\right).$$

**Figure 24:**
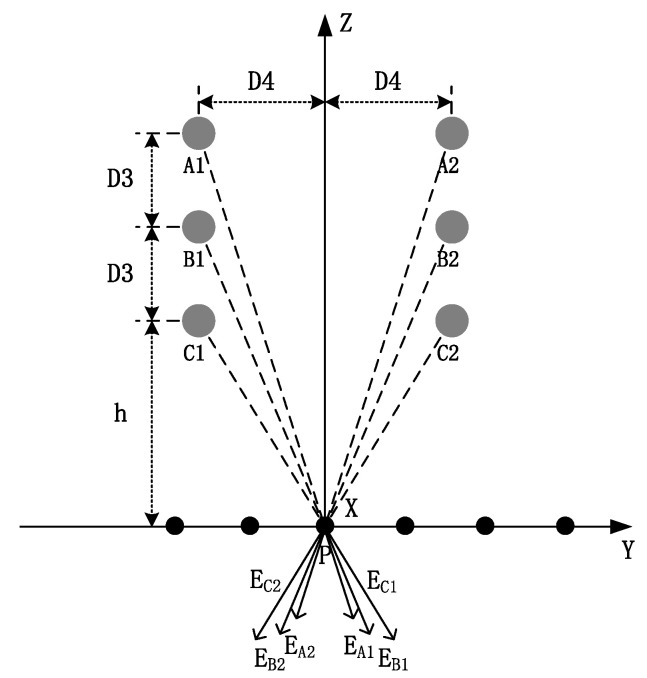
Double-back vertical arrangement of a three-phase wire.

If the verticality of the three-phase wire is not equal, the vertical components of the electric fields at P1 and P2 are:

(43) $$E=\frac{\lambda }{2\pi \varepsilon_{0}}\left(\frac{h_{A}}{h_{A}{}^{2}+D4^{2}}+\frac{h_{B}}{h_{B}{}^{2}+D4^{2}}+\frac{h_{C}}{h_{C}{}^{2}+D4^{2}}\right),$$

(44) $$E_{1}=\frac{\lambda }{2\pi \varepsilon_{0}}\left(\frac{h_{A}}{h_{A}{}^{2}+{\left(D4+Y_{0}\right)}^{2}}+\frac{h_{B}}{h_{B}{}^{2}+{\left(D4+Y_{0}\right)}^{2}}+\frac{h_{C}}{h_{C}{}^{2}+{\left(D4+Y_{0}\right)}^{2}}\right),$$

(45) $$E_{2}=\frac{\lambda }{2\pi \varepsilon_{0}}\left(\frac{h_{A}}{h_{A}{}^{2}+{\left(D4+Y_{0}\right)}^{2}}+\frac{h_{B}}{h_{B}{}^{2}+{\left(D4+2Y_{0}\right)}^{2}}+\frac{h_{C}}{h_{C}{}^{2}+{\left(D4+2Y_{0}\right)}^{2}}\right),$$

where *Y* refers to the horizontal distance from the measurement point on the ground to the wire. The horizontal distances are *D*4, *D*4 + *Y*_0_, and *D*4 + 2*Y*_0_ (Figure 23). Figure 24 depicts the double-back vertical arrangement of a three-phase wire.

The electric field at the ground measurement point is equal to the superposition of the electric fields generated by the three-phase conductive lines on both sides of the three-phase wires that are distributed with a dual vertical arrangement. The horizontal distance of the wires between the two sides is 2*D*4 and the valid amplitudes of the voltage passes are the same (Figure 24).

In this case, the spatial field of the parallel measurement points is on the horizontal ground. Six measurement points are selected if the vertical values of the dual-round wires are considered differently. The measured electric fields at each point are similar to those in the unilateral vertical arrangement, forming a measurement array. This characteristic can be expressed as:

(46) $$\left(\begin{array}{c} S_{A1}\\ S_{B1}\\ S_{C1}\\ S_{A2}\\ S_{B2}\\ S_{C2} \end{array}\right)=\left(\begin{array}{ccc} a_{11} & \cdots & a_{16}\\ \vdots & \ddots & \vdots \\ a_{61} & \cdots & a_{66} \end{array}\right)\left(\begin{array}{c} E_{1}\\ E_{2}\\ E_{3}\\ E_{4}\\ E_{5}\\ E_{6} \end{array}\right),$$

(47) $$F_{n}=\left(\begin{array}{ccc} a_{11} & \cdots & a_{16}\\ \vdots & \ddots & \vdots \\ a_{61} & \cdots & a_{66} \end{array}\right),$$

where *n* = 6.

If the sags of the wires on both sides are considered equal, the electric field only needs to be measured at one point to determine the sag, such as at P. According to symmetry, its electric field is given by:

(48) $$E=\frac{2\lambda }{2\pi \varepsilon_{0}}\left(\frac{h}{h^{2}+D4^{2}}+\frac{h+D3}{{\left(h+D3\right)}^{2}+D4^{2}}+\frac{h+2D3}{{\left(h+2D3\right)}^{2}+D4^{2}}\right).$$

**Figure 25:**
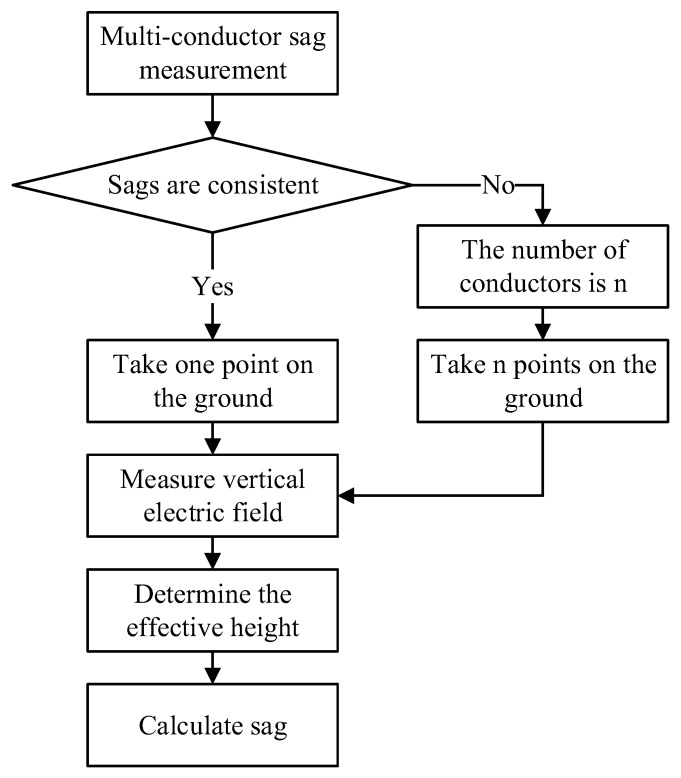
Multi-conductor sag measurement process.

The general calculation process for multi-conductor sags is shown in Figure 25.

If the vertical degree of each wire is considered consistent, then a position point can be selected on the ground. The effective wire height used to calculate the sag can be obtained by measuring the vertical component of the electric field. If the verticality of each wire is considered inconsistent, then the positions on the ground equal in number to that of the conductors are selected. The effective height of each wire is determined by measuring the vertical component of the electric field at each point to calculate the wires corresponding to each sag drooping value. The position of the measurement point is generally selected according to the condition parameters of the measurement matrix *F_n_* to reduce the ill-posed characteristics and calculation error of the inversion problem.

## 5. Conclusions

In this study, we conducted research on transmission line sag measurement and its simulation based on non-contact electric field sensing. The coupling relationships among the electric field, transmission line, and measurement point were established by analyzing the electric field distribution of the ideal horizontal and actual catenary conductors. In addition, an inverse sag calculation model was proposed.

The effects of the transmission line parameters, including the span length, tower height, and tower height difference, were studied according to the principle of sag measurement. It can be concluded that the effect of the tower height on the sag measurements was greater than that of the tower height difference, which was in turn greater than that of the span length. The line arrangement and sag measurement schemes of single- and dual-round lines were designed, and array research was performed for a multi-conductor line. Additionally, a multi-conductive sag measurement method was proposed. This method only requires the vertical component of the measured electric field and relative positions of the array and conductor to be determined; thus, transmission line sag measurement can be performed simply and efficiently.

The method proposed in this article has the advantages of adaptability and tolerance and allows for convenient operation. Not only does the theoretical aspect of the study agree with the finite element simulation to verify its feasibility, but it also considers multi-scenario distributions and indicates that the proposed theory can be applied to transmission lines of any structure. However, it should be noted that this method is still restricted in practical applications due to limitations in the actual environment, such as the effect of the tower on the line charge and the weather on the surrounding line. We can eliminate the effects of these factor variations on the results through field calibration and periodic multiple measurements. Specific implementation options, including measurement accuracy and performance assessment, will be considered in subsequent sensor design research. In general, this approach provides a wealth of reference information for the actual research, design, development, and application of transmission lines.

## Data Availability

Not applicable.
